# *STX1A* and Asperger syndrome: a replication study

**DOI:** 10.1186/2040-2392-5-14

**Published:** 2014-02-18

**Authors:** Jaroslava Durdiaková, Varun Warrier, Sharmila Banerjee-Basu, Simon Baron-Cohen, Bhismadev Chakrabarti

**Affiliations:** 1 Autism Research Centre, Department of Psychiatry, University of Cambridge, 18b Trumpington Road, Cambridge CB2 8AH, UK; 2 CLASS Clinic, Cambridgeshire and Peterborough NHS Foundation Trust (CPFT), Fulbourn, Cambridge CB21 5EF, UK; 3 Centre for Integrative Neuroscience and Neurodynamics, School of Psychology and Clinical Language Sciences, University of Reading, Reading RG6 6AL, UK; 4 Mindspec Inc, 9656 Blake Ln, Fairfax VA 22031, USA

**Keywords:** Asperger syndrome, Serotonergic system, Single nucleotide polymorphism, *STX1A*

## Abstract

**Background:**

Autism spectrum conditions (ASC) are a group of conditions characterized by difficulties in communication and social interaction, alongside unusually narrow interests and repetitive, stereotyped behaviour. Genetic association and expression studies have suggested an important role for the GABAergic circuits in ASC. Syntaxin 1A (*STX1A*) encodes a protein involved in regulation of serotonergic and GABAergic systems and its expression is altered in autism.

**Methods:**

In this study, the association between three single nucleotide polymorphisms (SNPs) (rs4717806, rs941298 and rs6951030) in *STX1A* gene and Asperger syndrome (AS) were tested in 650 controls and 479 individuals with AS, all of Caucasian ancestry.

**Results:**

rs4717806 (*P* = 0.00334) and rs941298 (*P* = 0.01741) showed a significant association with AS, replicating previous results. Both SNPs putatively alter transcription factor binding sites both directly and through other variants in high linkage disequilibrium.

**Conclusions:**

The current study confirms the role of *STX1A* as an important candidate gene in ASC. The exact molecular mechanisms through which *STX1A* contributes to the etiology remain to be elucidated.

## Background

Autism spectrum conditions (ASC) are characterized by difficulties in social interaction and impaired communication, alongside unusually narrow interests and repetitive, stereotyped behaviour (DSM-IV). Asperger syndrome (AS), a subcategory of ASC, differs from other autism conditions by lack of any delay in language and cognitive development [1]. ASC has a prevalence of 1 in 100 [2] and heritability of around 80% [3]. Several genes involved in neural transmission, and in particular GABAergic neurotransmission, have been implicated in ASC [4-7].

Syntaxin 1A (STX1A) is an important component of the GABAergic system, due to its involvement in the transport of glutamate and its conversion to gamma-aminobutyric acid (GABA) [8]. The protein STX1A is expressed presynaptically and is involved in neurotransmitter release and synaptic vesicle docking and thus is an essential factor for neurotransmission. It also interacts with serotonin and alters its subcellular localization and expression [9]. Atypical function in both serotonergic and GABAergic neurotransmission systems has been reported in autism [10]. GABAergic signaling during embryonic development contributes to the excitatory/inhibitory signaling balance in neural networks. Dysregulation of GABAergic system during development is thought to account for some of the sensory sensitivity in people with ASC [6].

**Figure 1:**
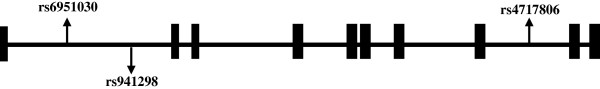
**Genomic structure of *STX1A *and the location of tested SNPs.** Exons are indicated by boxes; introns are indicated by lines; SNP positions are denoted by arrows (http://www.genome.ucsc.edu).

The gene *STX1A* consists of 10 exons (Figure 1) spanning a 20.42 kb region (http://www.genome.ucsc.edu). It lies on 7q11.23, a locus that has been reported to be duplicated in some individuals with autism, and associated with deletions in Williams syndrome [11]. A possible role for *STX1A* in the development of autism has previously been suggested. Three single nucleotide polymorphisms (SNPs) (rs2293485 and rs4717806 in the Caucasian population, and rs69510130 in the Japanese population) are nominally associated with high functioning autism (HFA) [12,13]. rs4717806 has also been related to early developmental differences in people with autism [12]. Another SNP, rs1569061, is nominally associated with ASC in a Caucasian population [11]. Additionally, *STX1A* mRNA expression is higher in lymphocytes of people with HFA [12], and lower in the anterior cingulated gyrus in post-mortem brain tissue [13], compared to matched controls. This makes *STX1A* an interesting candidate gene for ASC. In this study, we examined the association of three SNPs in *STX1A* with AS, in a Caucasian sample, in an effort to replicate previous findings.

## Methods

All individuals enrolled in the current study were adults of Caucasian origin from the same geographic region (the United Kingdom). Participants were asked to complete an online version of the Autism Spectrum Quotient (AQ) test [14], which is a measure of autistic traits. A score of 32 and above is an excellent predictor of ASC [15], and the mean AQ score in the general population is 16.4 (SD = 6.3) [14]. Individuals with a clinical diagnosis of AS (n = 479; 97 females, 382 males) took part and achieved a mean AQ score of 35.8 ± 8.3. The control group comprised participants (n = 650; 367 females, 284 males) chosen to have an AQ score below 24, to ensure a balanced representation of individuals from both ends of the autistic trait continuum. The mean AQ score within the control population was 14.8 ± 5.2. Consent was obtained from all the participants. This study was approved by the University of Cambridge Psychology Research Ethics Committee and the NHS National Research Ethics Service.

Based on previously published data, three SNPs (rs4717806, rs941298, rs6951030) were selected and analyzed in this study. rs4717806 and rs69510130 have been reported to be nominally associated with HFA in the Caucasian and Japanese population, respectively [12,13]. The third SNP, rs941298, is in high linkage disequilibrium (LD) with rs4717806 (R^2^ = 0.96) and in moderate LD with an exonic SNP, rs2293485 (R^2^ = 0.67), which is nominally associated with HFA in the Caucasian population [12]. LD values between SNPs of interest in the HapMap CEPH European samples of the Utah Residents with Northern and Western European Ancestry (CEU) population data were calculated using SNAP (http://www.broadinstitute.org/mpg/snap/). The minor allele frequency (MAF) for the tested SNPs was above 0.05 in the CEPH CEU population as calculated from the dbSNP database (http://www.ncbi.nlm.nih.gov/projects/SNP/). LD plots for the sample studied were created using Haploview [16].

DNA was extracted from buccal swabs and anonymized. SNP genotyping was performed using TaqMan SNP Genotyping Assays (Applied Biosystems Inc., CA, USA) according the previously described protocol [17]. Allelic association testing was performed using Plink v1.07 (http://pngu.mgh.harvard.edu/~purcell/plink/) [18]. Bonferroni correction was performed to correct for multiple SNPs tested after evaluating the number of completely independent SNPs and determination of a new alpha value using SNPSpD (http://gump.qimr.edu.au/general/daleN/SNPSpD/) [19]. This was done as Bonferroni correction is over-corrective when the SNPs are in LD. *P* values were reported as significant if they were below the alpha threshold.

Functional annotation was performed using Haploreg (http://www.broadinstitute.org/mammals/haploreg/haploreg.php) [20], SNPnexus (http://snp-nexus.org/) [21], and F-SNP (http://compbio.cs.queensu.ca/F-SNP/) [22].

## Results

**Figure 2:**
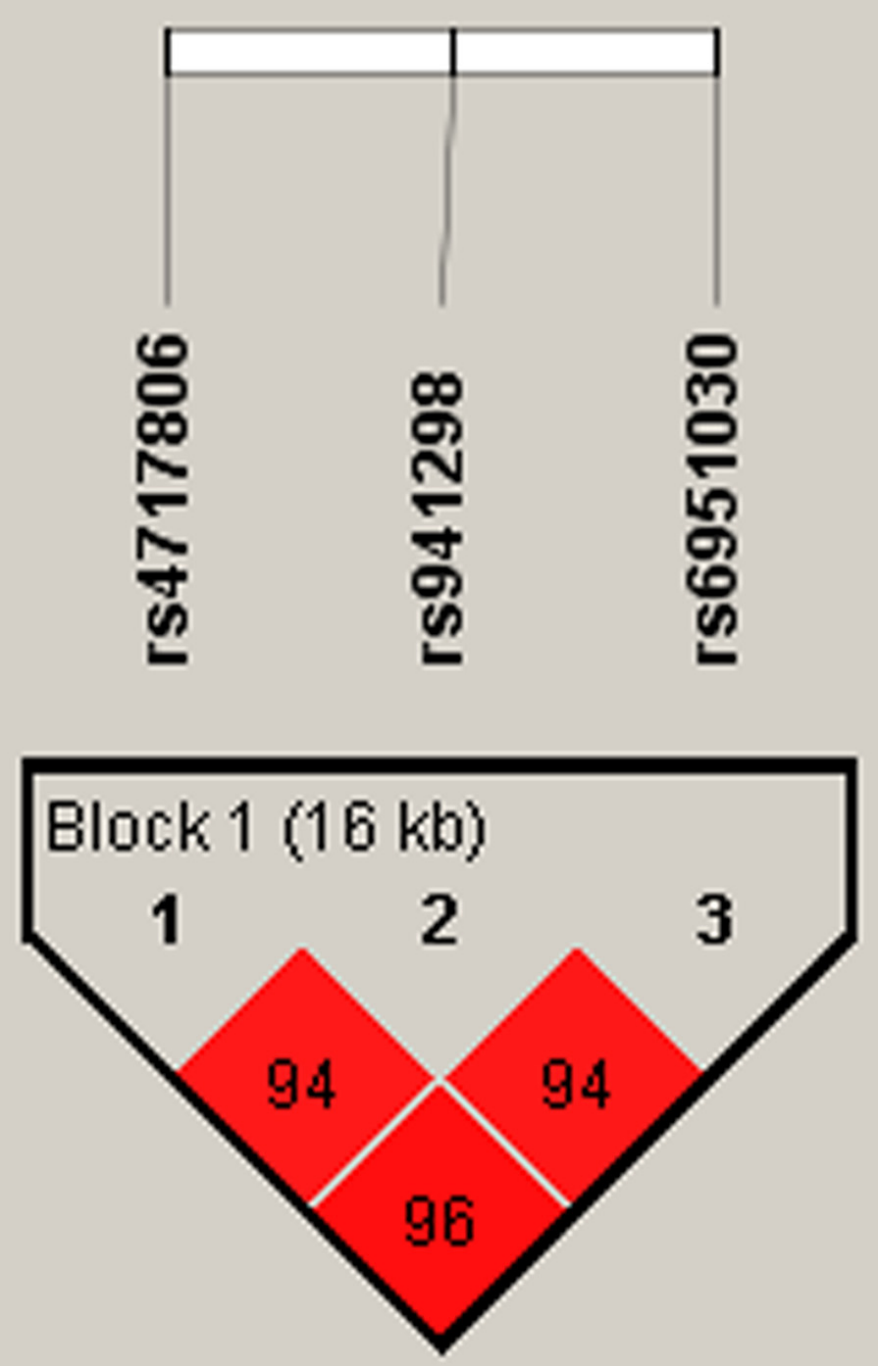
**LD structure of *STX1A *for AS case-control dataset.** LD structure of the region investigated in the AS case-control dataset, as plotted on Haplotype is shown. Three SNPs which were genotyped have been plotted; they fall into one LD block.

In the association analyses, rs4717806 (*P* = 0.003) and rs941298 (*P* = 0.017) showed significant association with AS. These results remained significant after correcting for multiple testing. The estimated average number of independent loci was 2. The SNPSpD determined threshold *P* value was 0.023. None of the SNPs deviated from the Hardy-Weinberg equilibrium at *P* < 0.05. Genotyping rate was above 98%. The test statistics are summarized in Table 1. In our sample, all three SNPs are part of a common LD block as calculated by Haploview (Figure 2).

**Table 1:** Single SNP association analyses

**dbSNp ID**	**Alleles**^**a**^	**MAF**^**b**^	**F_A**^**c**^	**F_U**^**d**^	**CHISQ**^**e**^	**Odds ratio**	***P *** **value**^**f**^	**Alpha**^**g**^
rs6951030	T/G	0.18	0.19	0.17	2.40	1.24	0.06	0.023
rs4717806	T/A	0.33	0.29	0.36	9.49	0.76	**0.003**	0.023
rs941298	G/A	0.33	0.29	0.35	6.09	0.80	**0.017**	0.023

^a^Common allele is listed first.

^b^Calculated by Plink v1.07 in analysed sample.

^c^The frequency of this variant in cases.

^d^The frequency of this variant in controls.

^e^The χ^2^ statistic for this test (1 df).

^f^Computed on the basis of likelihood ratio test.

^g^Determined after evaluating the number of completely independent SNPs using SNPSpD.

## Discussion

In this study, three SNPs in *STX1A* were tested for association with AS to replicate findings previously reported in the literature. We found that two of these SNPs (rs 4717806 and rs941298) were significantly associated with a clinical diagnosis of AS. The odds ratio for the two significant SNPs was below 0, indicating the protective role of the minor allele. rs4717806 and rs69510130 have been reported to be nominally associated with HFA in previous reports [12,13]. The direction of effect is in agreement with our findings. Both SNPs have been under-transmitted from non-affected parents to affected probands. rs941298 is in high LD with rs4717806 (R^2^ = 0.96) and in moderate LD with exonic rs2293485 (R^2^ = 0.67), which is also nominally associated with HFA [12].

We identified significant association between rs4717806 and AS, supporting previously published data [12]. rs941298 was also significantly associated with AS in the current study. We were not able to replicate the nominal association between rs69510130 and HFA [13] in our sample. However, rs69510130 was nominally significant in a Japanese sample in the previous study, whereas we tested for association in a Caucasian sample.

Both SNPs (rs4717806, rs941298) that showed significant association with AS in our study are intronic SNPs. rs4717806 lies in intron 9 and rs941298 lies in intron 2; they are 8,786 bp apart, in high LD in our sample (R^2^ = 0.92), and part of a common LD block as calculated by Haploview (Figure 2). Both the SNPs alter various transcription binding sites both directly and through other SNPs in LD (http://www.broadinstitute.org/mammals/haploreg/). There are no copy number polymorphisms or miRNA binding sites associated with either of the SNPs (http://www.snp-nexus.org/). rs4717806 is highly conserved across species according to F-SNP. It is likely that the expression of *STXA1* is regulated by these SNPs along with additional cis- and trans-acting factors.

While this study replicates the results from previous studies [12,13], it is different in that we focused on AS, rather than HFA or classic autism. Further, the previous studies used Transmission Disequilibrium Tests to identify over-transmitted alleles in family trios of individuals with ASC and HFA [12]. The current study design is population based and with a moderate sample size; hence, these results need to be replicated in a larger sample. It is worth mentioning that females are overrepresented in the control sample compared to the AS group. Men typically score higher than women in the self-reported AQ test [14], which may account for the lower AQ score describing the control population. In the sex-stratified analyses, none of the SNPs are significant after Bonferroni correction indicating that the over-representation of females in controls is not driving the association (data not shown).

Additionally, only three SNPs were tested, chosen specifically to replicate previous results. The high LD between rs4717806 and rs941298 must be taken into account when interpreting the results. It is likely that both the SNPs are tagging one single causative locus, which is reflected in the significant *P* values of the two SNPs. rs941298 would not remain significant if traditional Bonferroni correction was applied. A single gene is unlikely to have a major effect in complex conditions like autism, and many other genes are likely to contribute to the phenotype. This study, in combination with previous studies, provides evidence for *STX1A* as involved in the etiology of ASC. Further cellular, molecular, and neurocognitive studies are required to elucidate the role of this gene in ASC.

## Conclusions

rs4717806 (*P* = 0.00334) and rs941298 (*P* = 0.01741) showed significant association with AS, replicating findings in previous studies in the same direction. Both SNPs putatively alter transcription factor binding sites both directly and through other variants in high linkage disequilibrium. The current study confirms the role of *STX1A* as an important candidate gene in ASC. The exact molecular mechanisms through which *STX1A* contributes to the etiology remain to be elucidated.

## Abbreviations

AQ: Autism spectrum quotient; AS: Asperger syndrome; ASC: Autism spectrum conditions; CEU: European samples of Utah Residents with Northern and Western European Ancestry, GABA, Gamma-aminobutyric acid; HFA: High-functioning autism; LD: Linkage disequilibrium; MAF: Minor allele frequency; SNP: Single nucleotide polymorphism; STX1A: Syntaxin 1A.

## Competing interests

The authors declare that they have no competing interests.

## Authors’ contributions

BC and SBC co-designed the study. SBC obtained funding for the study. SBB suggested the mapping of *STX1A* as a relevant candidate gene. VW and JD conducted the analysis. JD wrote the first draft of the paper revised by all authors. All authors read and approved the final manuscript.
